# Obesity and sarcopenic obesity characterized by low-grade inflammation are associated with increased risk for major depression in women

**DOI:** 10.3389/fnut.2023.1222019

**Published:** 2023-09-28

**Authors:** Julie A. Pasco, Michael Berk, Brenda Penninx, Natalie K. Hyde, Kara L. Holloway-Kew, Emma C. West, Mark A. Kotowicz, Kara B. Anderson, Adrienne O’Neil, Pamela G. Rufus-Membere, Lana J. Williams

**Affiliations:** ^1^Deakin University, IMPACT – Institute for Mental and Physical Health and Clinical Translation, Barwon Health, Geelong, VIC, Australia; ^2^Department of Medicine–Western Health, The University of Melbourne, St. Albans, VIC, Australia; ^3^Department of Preventive Medicine, Monash University, Melbourne, VIC, Australia; ^4^The Florey Institute of Neuroscience and Mental Health, Parkville, VIC, Australia; ^5^Orygen, The National Centre of Excellence in Youth Mental Health, Parkville, VIC, Australia; ^6^Department of Psychiatry, Amsterdam Neuroscience and Amsterdam Public Health Research Institute, VU University Medical Centre, Amsterdam, Netherlands

**Keywords:** depression, obesity, sarcopenic obesity, high-sensitivity C-reactive protein, immunometabolic dysregulation, major depressive disorder, inflammation, mental disorders

## Abstract

**Background:**

We aimed to determine women’s risk of major depressive disorder (MDD) in relation to obesity phenotypes characterized by levels of circulating high-sensitivity C-reactive protein (hsCRP).

**Methods:**

This population-based retrospective cohort study comprised 808 women (ages 20–84 y) recruited 1994–1997 and followed for a median 16.1 y (IQR 11.9–16.8). At baseline, body fat and lean tissue mass were measured by whole body dual-energy x-ray absorptiometry (DXA). Obesity was identified as high fat mass index (>12.9 kg/m^2^), body fat percentage (≥35%) and body mass index (≥30 kg/m^2^); sarcopenic obesity referred to a high ratio fat mass/fat-free mass (≥0.80). Systemic inflammation was operationalized as serum hsCRP concentration in the upper tertile (>2.99 mg/L). Obesity phenotypes were: non-obese + lowCRP, non-obese + highCRP, obese + lowCRP, and obese + highCRP. During follow-up, the Structured Clinical Interview for DSM-IV-TR (SCID-I/NP) was used to identify lifetime history of MDD and age of onset. Poisson regression models were used to estimate the MDD rate for each obesity phenotype during follow-up. Demographic, health and lifestyle factors were tested as potential confounders.

**Results:**

During 11,869 p-y of follow-up, 161 (19.9%) women experienced an MDD episode. For obesity phenotypes based on fat mass index, models adjusted for baseline age and prior MDD, and non-obese + lowCRP as reference, RR for non-obese + highCRP was 1.21 (95% CI 0.80, 1.82), obese + lowCRP 1.46 (0.86, 2.47) and obese + highCRP 1.56 (1.03, 2.37). Patterns were similar for obesity by body fat percentage, body mass index and sarcopenic obesity.

**Conclusion:**

Consistently across different obesity definitions, this longitudinal study reports that women with both obesity and systemic inflammation are at increased risk of subsequent MDD. Future research should examine whether tackling this metabolically unhealthy obesity type – through, for example, lifestyle or medication approaches – can reduce depression risk.

## Introduction

Exacerbated by unhealthy lifestyles and other correlates of an obesogenic environment, obesity is a condition defined as “abnormal or excessive fat accumulation that may impair health” (1). It is characterized by large amounts of adipose tissue, an active endocrine organ (2) that can produce pro-inflammatory cytokines (3). Obesity is often comorbid with and bidirectionally linked to depression (4, 5). Together they increase the risk for cardiovascular and metabolic disease (6), partly via shared risk factors such as poor diet and physical inactivity driving common pathophysiological pathways including low-grade inflammation (7, 8). The concept of adiposity-driven inflammation in depression is supported by exemplar evidence from a cohort study that levels of C-reactive protein (CRP), which are indicative of systemic inflammation, account for approximately 20% of obesity-related longitudinal increases in depression scores (9), and that following gastric bypass surgery for patients with obesity, reductions in inflammatory markers CRP and interleukin-6 accompany weight loss and correlate with improved depressive symptoms (10).

However, not all individuals with obesity will develop depression; the relationship is complex (5). Further, some obese individuals appear to be metabolically healthy (11) whereas others are metabolically unhealthy, giving rise to the concept of metabolically healthy and unhealthy obesity. These phenotypes and corresponding metabolically healthy and unhealthy non-obesity, have been investigated mainly in relation to cardiometabolic risk (11, 12) but not much research has been undertaken in relation to mental health and depression, in particular.

Also understudied is the risk for depression conferred by effects of obesity coupled with low skeletal muscle mass, a composite state known as sarcopenic obesity (13, 14). While sarcopenic obesity describes obese individuals who also have sarcopenia, such that excessive body fat co-exists with low skeletal muscle mass and/or performance, currently there is no consensus for defining sarcopenic obesity. The condition is often characterized by sedentary lifestyles and aging where further loss of skeletal muscle might cause fat gain and vice versa (15). In sarcopenic obesity, excessive fat accumulates in skeletal muscle in a similar and parallel way to excessive fat accumulation in the liver, as seen in non-alcoholic fatty liver disease (16, 17). These ectopic fat deposits are associated with insulin resistance and cause an increased production and release of inflammatory factors and adipokines into the circulation, worsening low-grade inflammation (8, 18) and potentially driving metabolically unhealthy obesity.

Metabolic abnormality can be defined by the presence of the metabolic syndrome or its components (visceral fat accumulation, hypertension, dyslipidemia, hyperglycemia) (19); however, in this study we considered low grade inflammation as a marker of metabolic health. Previously, it has been identified that elevated CRP concentrations are comparable for individuals with metabolically unhealthy obesity and non-obesity (20). However, to our knowledge no studies have approached the link between metabolically unhealthy obesity and depressive morbidity by considering obesity associated inflammation rather than metabolic syndrome. We hypothesized that individuals with obesity and raised systemic inflammation would be at increased risk for major depressive disorder (MDD) over time. Our aim was to determine the risk of MDD in relation to obesity phenotypes characterized by levels of circulating high-sensitivity C-reactive protein (hsCRP).

## Materials and methods

### Study design and participants

This retrospective cohort study is part of the Geelong Osteoporosis Study, a population-based study involving women from the Barwon Statistical Division in south eastern Australia (21). An age-stratified sample of 1494 women aged 20–94 years was generated during the period from 1994 to 1997 for describing the epidemiology of osteoporosis and identifying risk factors for fracture. The Geelong Osteoporosis Study has since expanded to examine other metabolic and non-communicable disorders as well as mental disorders. Participants were selected at random from the Commonwealth electoral roll and invited by mail to participate; registration with the Australian Electoral Commission is compulsory for adults aged 18 years and over, so the electoral roll provides a comprehensive sampling frame. The sole inclusion criterion was a listing on the electoral roll as a resident of the Barwon Statistical Division and participants were excluded if they had been living in the region for less than 6 months and/or were unable to provide informed, written consent; participation was 77% and the majority (98%) of the sample was white. In this study, participants were followed after baseline for a median of 16.1 years (interquartile range, IQR, 11.9–16.8).

Baseline assessment included measures of body composition and collection of blood samples, as well as questionnaire and sociodemographic data. At the 10-year follow-up phase, 857 women participated in a psychiatric interview and of these, 820 women also provided baseline data required for this analysis and were thus eligible for this study. After the 10-year psychiatric assessment, 607 of the 820 women were re-assessed 5 years later. Written, informed consent was obtained from all participants. The Human Research Ethics Committee at Barwon Health approved the study (92/01).

### Data

#### Body composition

Body mass was measured to ±0.1 kg using electronic scales, standing height was measured to ± 0.001 m using a wall-mounted Harpenden stadiometer and body mass index calculated as body mass/height^2^ (kg/m^2^). To define obesity by body mass index criteria, we used values ≥ 30 kg/m^2^ (22). Body composition was measured by whole body dual-energy x-ray absorptiometry (DXA) using a Lunar DPX-L densitometer (software version 1.31; Lunar Madison, WI, USA) to identify body fat mass (FM), lean mass and bone mass. Fat mass index was calculated as fat mass expressed relative to height (kg/m^2^), and body fat percentage as fat mass expressed as a percentage of total tissue mass. Obesity was identified fat mass index >12.9 kg/m^2^ (23) and body fat percentage ≥ 35% (24, 25). With fat-free mass referring to the sum of lean and bone mass, sarcopenic obesity was identified by the ratio fat mass/fat-free mass ≥ 0.80 (26), as this threshold delineates health risks associated with excessive fat (which confers metabolic load) coincident with diminished muscle (which confers metabolic capacity) (13). Long-term stability of the DXA was confirmed by scanning an anthropomorphic phantom three times a week, and physical and mental health assessments were conducted by different personnel, all of whom were appropriately trained.

#### Biomarker

Blood samples were collected in the morning, following an overnight fast, and stored at −80°C until batch analysis. Serum hsCRP was measured by the Roche immunoturbidimetric “CRP” and “C-reactive protein (latex) high sensitivity” methods, as previously described (27, 28). Samples were first analyzed using the high sensitivity assay, which has a range of 0.1–20 mg/L, and those with results >20 mg/L were reanalyzed using the CRP assay, with a range of 3–480 mg/L. Long-term inter-assay coefficients of variation were <10% at 1 mg/L and <5% at 5 mg/L. High hsCRP was identified by values in the upper tertile of the hsCRP distribution (hsCRP > 2.99 mg/L).

#### Other exposure data

Details of lifestyle behaviors, medication use and disease status were documented by self-report. In this analysis, smoking refers to current tobacco use, and alcohol use recognized if alcohol was consumed daily. Mobility was described as “very active” or “active” if vigorous or light exercise was performed regularly; individuals were otherwise classified as sedentary (21). Medication use was captured by questionnaire and confirmed where possible by cross-checking with medication containers or lists brought to clinical assessment by participants. Medications used regularly at the time of assessment included antidepressants and agents that are recognized as affecting CRP levels, namely non-steroidal anti-inflammatory drugs (NSAIDs, including aspirin), oral glucocorticoids, antihyperlipidaemics, angiotensin-converting enzyme (ACE) inhibitors, beta-blockers, antihyperglycemics, anticoagulants and vitamin E (29). A history of cancer, pernicious anemia, rheumatoid arthritis and lupus referred to ever exposure to these diseases. Socio-economic status was ascertained using Socio-Economic Index for Areas scores based on census data from the Australian Bureau of Statistics (1996) and used to derive an Index of Relative Socio-Economic Disadvantage (IRSD) that was grouped into quintiles of IRSD for the study region.

#### MDD

The Structured Clinical Interview for DSM-IV-TR Research Version, Non-patient edition (SCID-I/NP) was used to identify women with a lifetime history of MDD and to determine age of onset (30). The psychiatric interviews were conducted by trained personnel with a tertiary qualification in Psychology; they were blinded to body composition, biochemical and questionnaire data. Of 820 participants with complete data, 301 were identified with a lifetime history of MDD; 161 with at least one MDD event post-baseline, 128 with prior (pre-baseline) MDD and 12 were excluded as MDD onset occurred within 12 months of baseline. Thus, 808 women were eligible for analyses.

### Statistics

Using obesity defined by fat mass index > 12.9 kg/m^2^, body fat percentage ≥ 35%, body mass index ≥ 30 kg/m^2^ and high CRP defined as hsCRP > 2.99 mg/L, participants were grouped into four phenotypes based on their body composition and inflammatory status: (1) non-obesity with low inflammation (non-obese + lowCRP). (2) non-obesity with high inflammation (non-obese + highCRP); (3) obesity with low inflammation (obese + lowCRP); (4) obesity with high inflammation (obese + highCRP). Similarly, using sarcopenic obesity defined as fat mass/fat-free mass ≥ 0.80 and high CRP as hsCRP > 2.99 mg/L, the four obesity phenotypes involving the presence/absence of sarcopenic obesity (SO) and high/low inflammation were (1) non-sarcopenic obesity with low inflammation (nonSO + lowCRP). (2) non-sarcopenic obesity with high inflammation (nonSO + highCRP); (3) sarcopenic obesity with low inflammation (SO + lowCRP); (4) sarcopenic obesity with high inflammation (SO + highCRP).

Standard descriptive statistics were used to describe participant characteristics, and intergroup differences were identified using Student *t*-tests or ANOVA for continuous parametric variables, the Mann–Whitney or Kruskal–Wallis test for continuous non-parametric variables, and the Chi-square test for discrete variables. Histograms were used to inspect the distribution of continuous data. Multivariable Poisson regression models were developed to estimate the rate ratio (RR) of at least one MDD event during follow-up for each obesity phenotype, after testing other exposure variables (age, prior MDD, and demographic, health and lifestyle factors including smoking, alcohol consumption, physical activity, socioeconomic status, use of antidepressants and other medications that affect CRP levels, and a history of inflammatory diseases) as potential confounders. Backward elimination was used to identify factors that did not contribute to the model; non-significant factors (*p* < 0.05) were sequentially removed while constructing the final models. The reference group was changed to identify differences in MDD rates between other obesity subgroups. RRs were expressed together with 95% confidence intervals (CIs). Statistical analyses were performed using Stata (release 17, StataCorp, College Station, TX) and Minitab (version 16; Minitab, State College, PA).

## Results

Table 1 lists summary characteristics for all participants. Table 2 lists characteristics according to obesity phenotypes.

**TABLE 1:** Participant characteristics at baseline.

	*n* = 808
Age (y)	47.6 (35.9–60.7)
Serum hsCRP (mg/L)	1.89 (0.89–3.98)
Body habitus	
Height (m)	1.61 (± 0.06)
Body mass (kg)	69.2 (± 13.9)
Fat mass (kg)	26.9 (± 10.2)
Lean mass (kg)	38.9 (± 4.3)
Fat mass index (kg/m^2^)	10.4 (± 3.9)
Body fat percentage (%)	38.2 (± 8.0)
Body mass index (kg/m^2^)	26.6 (± 5.3)
Fat mass/fat-free mass	0.65 (± 0.21)
Fat mass index > 12.9 kg/m^2^	195 (24.1%)
Body fat percentage ≥ 35%	556 (68.8%)
Body mass index > 30 kg/m^2^	200 (24.8%)
Fat mass/fat-free mass > 0.80	237 (29.3%)
Socioeconomic status	
Quintile 1 (low)	138 (17.1%)
Quintile 2	165 (20.4%)
Quintile 3	175 (21.7%)
Quintile 4	149 (18.4%)
Quintile 5	181 (22.4%)
Lifestyle behaviors	
Current smoker	117 (14.5%)
Alcohol daily	63 (7.8%)
Physical activity	
Very active	103 (12.8%)
Active	529 (65.5%)
Sedentary	176 (21.8%)
Medications	
Antidepressants	32 (4.0%)
NSAIDs	139 (17.2%)
Oral glucocorticoids	7 (0.9%)
Antihyperlipidaemics	24 (3.0%)
ACE inhibitors, ARBs	57 (7.1%)
Beta-blockers	50 (6.2%)
Anti-hyperglycemics	9 (1.1%)
Anti-coagulants	13 (1.6%)
Vitamin E	10 (1.2%)
Disease history	
Prior MDD	128 (15.8%)
Cancer	45 (5.6%)
Pernicious anemia	22 (2.7%)
Rheumatoid arthritis	2 (0.2%)
Lupus	2 (0.2%)

Data are shown as mean (± SD), median (IQR) or number (%). hsCRP, high-sensitivity C-reactive protein; NSAIDs, non-steroidal anti-inflammatory drugs (including aspirin); ACE, inhibitors angiotensin-converting enzyme inhibitors; ARBs, angiotensin receptor blockers; MDD, major depressive disorder.

**TABLE 2:** Baseline characteristics for participants by obesity phenotype.

	Obesity phenotype				
	**Non-obese + lowCRP**	**Non-obese + highCRP**	**Obese + lowCRP**	**Obese + highCRP**	**P for difference^†^**
**Fat mass index > 12.9 kg/m^**2**^**	***n* = 462**	***n* = 151**	***n* = 77**	***n* = 118**	
Age (y)	45.4 (35.1–59.8)	50.5 (34.5–62.9)	52.2 (39.0–62.0)	37.6 (39.1–60.2)	0.100
Serum hsCRP (mg/L)	1.00 (0.57–1.77)	4.89 (3.79–7.40)	1.70 (1.20–2.33)	6.24 (4.39–10.23)	<0.001
Body habitus					
Height (m)	1.62 (± 0.06)	1.61 (± 0.06)	1.60 (± 0.06)	1.60 (± 0.06)	0.001
Body mass (kg)	62.8 (± 8.3)	64.6 (± 8.7)	83.7 (± 9.3)	89.4 (± 13.5)	<0.001
Fat mass (kg)	21.8 (± 6.2)	24.7 (± 6.1)	39.1 (± 5.7)	42.0 (± 7.6)	<0.001
Lean mass (kg)	38.1 (± 3.9)	38.0 (± 3.8)	41.1 (± 4.2)	41.9 (± 4.8)	<0.001
Socioeconomic status					0.006
Quintile 1 (low)	66 (14.3%)	30 (19.9%)	12 (15.6%)	30 (25.4%)	
Quintile 2	84 (18.2%)	38 (25.2%)	18 (23.4%)	25 (21.2%)	
Quintile 3	104 (22.5%)	30 (19.9%)	22 (28.6%)	19 (16.1%)	
Quintile 4	85 (18.4%)	22 (14.6%)	14 (18.2%)	28 (23.7%)	
Quintile 5	123 (26.6%)	31 (20.5%)	11 (11.3%)	16 (13.6%)	
Lifestyle behaviors					
Current smoker	66 (14.3%)	26 (17.2%)	10 (13.0%)	15 (12.7%)	0.714
Alcohol daily	43 (9.3%)	13 (8.6%)	3 (3.9%)	4 (3.4%)	0.093
Physical activity					<0.001
Very active	77 (16.7%)	17 (11.3%)	4 (5.2%)	5 (4.2%)	
Active	309 (66.9%)	100 (66.2%)	49 (63.6%)	71 (60.2%)	
Sedentary	76 (16.5%)	34 (22.5%)	24 (31.2%)	42 (35.6%)	
Medications					
Antidepressants	16 (3.5%)	6 (4.0%)	3 (3.9%)	7 (5.9%)	-
Agent that lowers CRP*	120 (26.0%)	57 (37.8%)	31 (40.3%)	46 (39.0%)	0.002
Prior MDD	72 (15.6%)	25 (16.6%)	12 (15.6%)	19 (16.1%)	0.993
**Body fat percentage > 35%**	***n* = 210**	***n* = 42**	***n* = 329**	***n* = 227**	
Age (y)	39.6 (30.6–51.1)	36.9 (33.0–60.0)	52.0 (39.6–64.0)	50.4 (38.8–61.9)	<0.001
Serum hsCRP (mg/L)	0.74 (0.38–1.37)	4.50 (3.50–7.38)	1.29 (0.90–2.16)	5.53 (4.03–9.05)	<0.001
Body habitus					
Height (m)	1.63 (± 0.06)	1.62 (± 0.06)	1.27 (± 0.07)	1.60 (± 0.06)	0.001
Body mass (kg)	57.9 (± 6.4)	58.0 (± 6.8)	70.9 (± 10.6)	79.4 (± 15.2)	<0.001
Fat mass (kg)	16.5 (± 3.7)	17.4 (± 3.8)	29.2 (± 7.1)	35.1 (± 9.6)	<0.001
Lean mass (kg)	38.5 (± 3.7)	38.0 (± 3.8)	38.5 (± 4.2)	40.0 (± 4.8)	<0.001
Socioeconomic status					0.016
Quintile 1 (low)	32 (13.5%)	9 (18.4%)	46 (15.2%)	51 (23.2%)	
Quintile 2	39 (16.5%)	13 (26.5%)	63 (20.9%)	50 (22.7%)	
Quintile 3	61 (25.7%)	10 (20.4%)	65 (21.5%)	39 (17.7%)	
Quintile 4	37 (15.6%)	6 (12.2%)	62 (20.5%)	44 (20.0%)	
Quintile 5	68 (28.7%)	11 (22.5%)	66 (21.9%)	36 (16.4%)	
Lifestyle behaviors					
Current smoker	39 (16.5%)	10 (20.4%)	37 (12.3%)	31 (14.1%)	0.337
Alcohol daily	20 (8.4%)	2 (4.1%)	26 (8.6%)	15 (6.8%)	-
Physical activity					<0.001
Very active	61 (25.7%)	8 (16.3%)	20 (6.6%)	14 (6.4%)	
Active	148 (62.5%)	32 (65.3%)	210 (69.5%)	139 (63.2%)	
Sedentary	28 (11.8%)	9 (18.4%)	72 (23.8%)	67 (30.5%)	
Medications					
Antidepressants	6 (2.5%)	2 (4.1%)	13 (4.3%)	11 (5.0%)	-
Agent that lowers CRP*	33 (13.9%)	12 (24.5%)	47 (15.6%)	47 (21.4%)	0.077
Prior MDD	37 (13.5%)	9 (15.8%)	47 (17.7%)	35 (16.5%)	0.592
**Body mass index > 30 kg/m^**2**^**	***n* = 457**	***n* = 151**	***n* = 82**	***n* = 118**	
Age (y)	45.2 (34.8–59.5)	50.6 (34.6–62.8)	52.6 (40.0–63.5)	49.2 (37.4–60.2)	0.027
Serum hsCRP (mg/L)	1.00 (0.57–1.76)	4.86 (3.75–7.07)	1.83 (1.20–2.33)	6.33 (4.38–10.31)	<0.001
Body habitus					
Height (m)	1.62 (± 0.06)	1.61 (± 0.06)	1.60 (± 0.06)	1.61 (± 0.06)	0.003
Body mass (kg)	62.6 (± 8.0)	65.2 (± 8.1)	83.8 (± 8.8)	89.9 (± 13.0)	<0.001
Fat mass (kg)	21.7 (± 6.2)	24.8 (± 6.3)	38.4 (± 5.9)	41.9 (± 7.8)	<0.001
Lean mass (kg)	38.0 (± 3.8)	37.5 (± 3.7)	41.5 (± 4.0)	42.5 (± 4.3)	<0.001
Socioeconomic status					0.004
Quintile 1 (low)	64 (14.0%)	28 (18.4%)	14 (17.1%)	32 (27.1%)	
Quintile 2	83 (18.2%)	39 (25.8%)	19 (23.2%)	24 (20.3%)	
Quintile 3	104 (22.8%)	29 (19.2%)	22 (26.8%)	20 (17.0%)	
Quintile 4	83 (18.2%)	24 (15.9%)	16 (19.5%)	26 (22.0%)	
Quintile 5	123 (26.9%)	31 (20.5%)	11 (13.4%)	16 (13.6%)	
Lifestyle behaviors					
Current smoker	65 (14.2%)	23 (15.2%)	11 (13.4%)	18 (15.3%)	0.973
Alcohol daily	43 (9.4%)	15 (9.9%)	3 (3.7%)	2 (1.7%)	0.014
Physical activity					<0.001
Very active	78 (17.1%)	17 (11.3%)	3 (3.7%)	5 (4.2%)	
Active	303 (66.3%)	98 (64.9%)	55 (67.1%)	73 (61.9%)	
Sedentary	76 (16.6%)	36 (23.8%)	24 (29.3%)	40 (33.9%)	
Medications					
Antidepressant	16 (3.5%)	8 (5.3%)	3 (3.7%)	5 (4.2%)	-
Agent that lowers CRP*	118 (25.8%)	59 (39.1%)	33 (40.2%)	44 (37.3%)	0.001
Prior MDD	69 (15.1%)	28 (18.5%)	15 (18.3%)	16 (13.6%)	0.605
**Fat mass/fat-free mass > 0.80**	***n* = 435**	***n* = 136**	***n* = 104**	***n* = 133**	
Age (y)	44.8 (35.0–58.4)	49.7 (34.2–62.7)	52.8 (40.5–63.8)	50.0 (38.9–60.9)	0.013
Serum hsCRP (mg/L)	0.98 (0.53–1.71)	4.91 (3.80–8.34)	1.71 (1.13–2.29)	6.03 (4.17–9.12)	<0.001
Body habitus					
Height (m)	1.62 (± 0.06)	1.61 (± 0.06)	1.60 (± 0.07)	1.61 (± 0.06)	<0.001
Body mass (kg)	62.6 (± 8.43)	65.9 (± 9.4)	79.2 (± 11.1)	86.4 (± 14.9)	<0.001
Fat mass (kg)	21.3 (± 6.0)	24.2 (± 6.2)	36.6 (± 6.6)	40.6 (± 8.3)	<0.001
Lean mass (kg)	0.52 (± 0.14)	0.59 (± 0.13)	0.87 (± 0.11)	0.93 (± 0.14)	<0.001
Socioeconomic status					0.023
Quintile 1 (low)	59 (13.6%)	28 (20.6%)	19 (18.3%)	32 (24.1%)	
Quintile 2	82 (18.9%)	33 (24.3%)	20 (19.2%)	30 (22.6%)	
Quintile 3	98 (22.5%)	29 (21.3%)	28 (26.9%)	20 (15.0%)	
Quintile 4	81 (18.6%)	20 (14.7%)	18 (17.3%)	30 (22.6%)	
Quintile 5	115 (26.4%)	26 (19.1%)	19 (18.3%)	21 (15.8%)	
Lifestyle behaviors					
Current smoker	61 (14.0%)	26 (19.1%)	15 (14.4%)	15 (11.3%)	0.316
Alcohol daily	36 (8.3%)	11 (8.1%)	10 (9.6%)	6 (4.5%)	0.452
Physical activity					<0.001
Very active	77 (17.7%)	17 (12.5%)	4 (3.9%)	5 (3.8%)	
Active	289 (66.4%)	89 (65.4%)	69 (66.4%)	82 (61.7%)	
Sedentary	69 (15.9%)	30 (22.1%)	31 (29.8%)	46 (34.6%)	
Medications					
Antidepressant	14 (3.2%)	4 (2.9%)	5 (4.8%)	9 (6.8%)	0.267
Agent that lowers CRP*	117 (26.9%)	51 (37.5%)	34 (32.7%)	52 (39.1%)	0.017
Prior MDD	69 (15.9%)	21 (15.4%)	15 (14.4%)	23 (17.3%)	0.944

Data are shown as mean (± SD), median (IQR) or number (%). CRP, C-reactive protein; hsCRP, high sensitivity C-reactive protein; MDD, major depressive disorder. *Agents that lower CRP: oral glucocorticoids, NSAIDs (including aspirin), antihyperlipidaemics, ACE inhibitors’ ARBs, beta-blockers, anti-hyperglycemics, anticoagulants, vitamin E. p† represents the p for intergroup differences identified using ANOVA for continuous parametric variables, the Kruskal–Wallis test for continuous non-parametric variables, and the Chi-square test for discrete variables.

### Rate of MDD during follow-up

Among 808 participants, 161 (19.9%) experienced at least one MDD episode during 11,869 person-years of follow-up and 647 were MDD-free. Compared to participants without post-baseline MDD, those with MDD were younger [median (IQR), 41.3 (32.4–50.3) vs. 50.2 (37.0–62.7) y, *p* < 0.001], had higher mean fat mass index (11.0 ± 4.4 vs. 10.2 ± 3.8 kg/m^2^, *p* = 0.031), body fat percentage (39.4 ± 8.4 vs. 37.9 ± 7.9%, *p* = 0.049) and body mass index (27.4 ± 6.1 vs. 26.4 ± 5.1 kg/m2, *p* = 0.049), were more likely to be current smokers [n (%), 39 (24.2%) vs. 78 (12.1%), *p* < 0.001], and have a history of MDD [80 (49.7%) vs. 48 (7.4%), *p* < 0.001], and were less likely to consume alcohol daily [5 (3.1%) vs. 58 (9.0%), *p* = 0.013] and use medications known to reduce hsCRP [14 (8.7%) vs. 120 (18.6%), *p* = 0.003]. No other differences were detected. The rate of MDD during follow-up was 63.1 per 1,000 person-years (95% CI 54.1, 73.7).

### Inflammation

The distribution of hsCRP was positively skewed with a median value of 1.89 mg/L (IQR 0.89–3.98). Fifty-two of 808 (6.4%) participants with hsCRP values > 10 mg/L, concordant with an acute inflammatory response, were retained in analyses as no difference was observed in the proportions who developed MDD.

### Obesity phenotypes and MDD rate

#### Fat mass index

Obesity by fat mass index was identified in 195 (24.1%) participants as their fat mass index was > 12.9 kg/m^2^. The MDD rates were 12.5 (10.4, 15.1) per 1,000p-y for non-obesity, and 16.8 (12.6, 22.3) per 1,000p-y for obesity. In a model adjusted for age and prior MDD, the RR for MDD associated with obesity was 1.45 (95% CI 1.03, 2.04; *p* = 0.031).

Obesity phenotypes numbered 462 (57.2%) for non-obese + lowCRP, 151 (18.7%) for non-obese + highCRP, 77 (8.1%) for obese + lowCRP and 118 (14.6%) for obese + highCRP.

The MDD rates were 11.9 (95% CI 9.6, 14.8) per 1,000p-y for non-obese + lowCRP; 14.6 (95% CI 10.3, 20.6) per 1,000p-y for non-obese + highCRP; 15.2 (95% CI 9.4, 24.4) per 1,000p-y for obese + lowCRP; and 17.8 (95% CI 12.5, 25.3) per 1,000p-y for obese + highCRP.

#### Body fat percentage

Obesity by body fat percentage was identified in 556 (68.8%) participants as their body fat percentage was ≥ 35%. Rates of MDD were 10.4 (95% CI 7.6, 14.2) per 1,000p-y for non-obesity, and 15.0 (12.6, 18.0) per 1,000p-y for obesity. In a model adjusted for age and prior MDD, the RR for MDD associated with obesity was 1.48 (95% CI 1.02, 2.14; *p* = 0.040).

Obesity phenotypes numbered 210 (26.0%) for non-obese + lowCRP, 42 (5.2%) for non-obese + highCRP, 329 (40.7%) for obese + lowCRP and 227 (28.1%) for obese + highCRP.

MDD rates were 10.8 (95% CI 7.7, 15.1) per 1,000p-y for non-obese + lowCRP; 8.3 (95% CI 3.4, 19.9) per 1000 p-y for non-obese + highCRP; 13.4 (95% CI 10.5, 17.1) per 1,000p-y for obese + lowCRP; and 17.4 (95% CI 13.5, 22.5) per 1000p-y for obese + highCRP.

#### Body mass index

Obesity by body mass index was identified in 200 (24.8%) participants as their body mass index was ≥ 30 kg/m^2^. The MDD rate for the group with non-obesity was 12.9 (95% CI 10.7, 15.5) per 1000p-y, and for the group with obesity, 15.6 (95% CI 11.7, 20.8) per 1000p-y. In a model adjusted for age and prior MDD, the RR for MDD associated with obesity was 1.33 (95% CI 0.94, 1.88; *p* = 0.106).

There were 457 (56.6%) participants with non-obese + lowCRP, 151 (18.7%) with non-obese + highCRP, 82 (10.1%) with obese + lowCRP and 118 (14.6%) with obese + highCRP. For each obesity phenotype the MDD rates were 12.0 (95% CI 9.7, 15.0) per 1000p-y for non-obese + lowCRP; 15.6 (95% CI 11.1, 21.8) per 1000p-y for non-obese + highCRP; 14.1 (95% CI 8.8, 22.8) per 1000p-y for obese + lowCRP; and 16.5 (95% CI 11.5, 23.8) per 1000p-y for obese + highCRP.

#### Sarcopenic obesity

Sarcopenic obesity was identified in 237 (29.3%) participants as their fat mass/fat-free mass was ≥ 0.80. The MDD rates were 12.3 (10.2, 14.9) per 1000p-y for non-sarcopenic obesity, and 16.7 (12.9, 21.6) per 1000p-y for sarcopenic obesity. In a model adjusted for age and prior MDD, the RR for MDD associated with obesity was 1.47 (95% CI 1.06, 2.03; *p* = 0.021).

Obesity phenotypes numbered 435 (53.8%) for nonSO + lowCRP, 136 (16.8%) for nonSO + highCRP, 104 (12.9%) for SO + lowCRP and 133 (16.5%) for SO + highCRP.

The MDD rates were 11.9 (95% CI 9.5, 14.9) per 1000p-y for nonSO + lowCRP; 13.5 (95% CI 9.3, 19.7) per 1000p-y for nonSO + highCRP; 14.2 (95% CI 9.2, 21.7) per 1000p-y for SO + lowCRP; and 18.6 (95% CI 13.4, 25.8) per 1000p-y for SO + highCRP.

**FIGURE 1:**
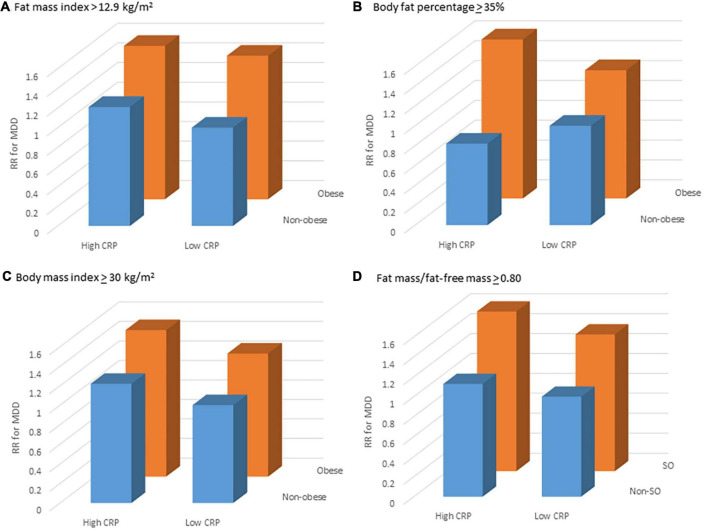
Relative rate (RR) for major depressive disorder (MDD) for different obesity phenotypes: non-obese + lowCRP (reference), non-obese + highCRP, obese + lowCRP, and obese + highCRP, where obesity has been identified by **(A)** fat mass index, **(B)** body fat percentage and **(C)** body mass index, and sarcopenic obesity by **(D)** fat mass/fat-free mass; and high levels of systemic inflammation (hsCRP > 2.99 mg/L). Point estimates are adjusted for age and prior MDD.

Using multivariable models and non-obese + lowCRP as the reference group, relative rates for MDD for obesity phenotypes are shown in Table 3. Irrespective of which definition was used for obesity, MDD rates did not differ between non-obese + lowCRP and non-obese + highCRP; in each case, while the highest MDD rate was observed for obese + highCRP, the rate difference between obese + lowCRP and obese + highCRP was not significant (*p* > 0.05). The same pattern was observed when sarcopenic obesity was considered in the four obesity phenotypes. Figure 1 shows data for obesity according to fat mass index, body fat percentage, body mass index, and sarcopenic obesity by fat mass/fat-free mass. All models were adjusted for baseline age and prior MDD. While some inter-group differences were observed for physical activity, and use of alcohol and medications known to lower CRP, there was a consistent pattern of SES differences across groups indicating lower than expected numbers in the obese + highCRP group with high SES and lower than expected numbers in the non-obese + lowCRP with low SES. Despite these differences, none of the other demographic, medication or lifestyle factors were identified as confounders in the Poisson regression models.

**TABLE 3:** Relative rate (RR) for major depressive disorder (MDD) for different obesity phenotypes.

	Obesity phenotypes	RR	Std error	*P*	95% CI
Fat mass index	Non-obese + lowCRP	Reference			
	Non-obese + highCRP	1.21	0.252	0.366	0.80, 1.82
	Obese + lowCRP	1.46	0.392	0.158	0.86, 2.47
	Obese + highCRP	1.56	0.332	0.034	1.03, 2.37
Body fat percentage	Non-obese + lowCRP	Reference			
	Non-obese + highCRP	0.82	0.392	0.675	0.32, 2.09
	Obese + lowCRP	1.29	0.280	0.241	0.84, 1.97
	Obese + highCRP	1.63	0.357	0.026	1.06, 2.50
Body mass index	Non-obese + lowCRP	Reference			
	Non-obese + highCRP	1.22	0.249	0.338	0.81, 1.82
	Obese + lowCRP	1.26	0.338	0.396	0.74, 2.13
	Obese + highCRP	1.50	0.326	0.060	0.98, 2.30
Sarcopenic obesity	nonSO + lowCRP	Reference			
	nonSO + highCRP	1.13	0.25	0.580	0.73, 1.76
	SO + lowCRP	1.37	0.34	0.207	0.84, 2.22
	SO + highCRP	1.62	0.33	0.018	1.09, 2.40

Models are adjusted for baseline age and prior MDD. CRP, C-reactive protein; SO, sarcopenic obesity.

## Discussion

In this retrospective cohort study of women followed for 16.1 years, the MDD rate for women with obesity was 1.5–1.6 fold higher compared to those without obesity. The MDD rate was greatest for women with obesity and high inflammation, and this pattern was independent of criteria for identifying obesity. No significant inter-group differences in MDD rates were observed for the other obesity phenotypes. Statistical models accounted for differences in age and prior MDD, yet patterns were not explained by the relatively low social economic status and sedentariness observed for women with obesity and high inflammation. This aligns with a previous observation that associations between immunometabolic dysregulation and depression appear to be somewhat independent of poor health behaviors and low socioeconomic status (31).

Evidence from epidemiological studies implicate bidirectional obesity-depression relationships (4). In a pooled analysis of eight cross-sectional studies involving over 30,000 men and women aged 15–105 years, measures included body mass index and metabolic risk factors (hypertension and biomarkers for an unfavorable metabolic profile) and depressive symptoms. Individuals with metabolically healthy obesity were at a slightly higher risk for depressive symptoms than non-obese individuals, but the greatest risk for depressive symptoms was observed for those with the metabolically unhealthy obesity phenotype (32). Thus, the association between obesity and risk of depression appeared to be partly dependent on metabolic health. Using similar criteria, obesity phenotypes were identified at baseline for older men and women drawn from the general population and enrolled in the English Longitudinal Study of Aging (ELSA) and followed over 2 years (20). Their results showed that, compared to the metabolically healthy non-obese reference group, those with metabolically unhealthy obesity had a greater risk for depressive symptoms, whereas the group with metabolically healthy obesity did not.

Two more recent, large studies utilized data from national health databases; both identified obesity phenotypes by body mass index and components of metabolic syndrome. One, a cross-sectional analysis of over nine million men and women from the Nationwide Inpatient Sample database in the USA, identified that an increased risk of obesity-related depression increased with the number of metabolic risk factors (33). In their study, depression included MDD and mood or dysthymic disorders with depressive features. Results showed that the metabolically unhealthy obesity phenotype had the highest risk for depression, metabolically healthy groups had the lowest risk, and the group with metabolically healthy obesity had intermediate risk. This pattern is similar to ours, except our intergroup differences were not all significant, potentially a consequence of our relatively small sample size. The other recent study involved longitudinal data from over three million men and women in the National Health Insurance Database of Korea. Depression in this study was identified as newly-diagnosed depression on health insurance data or antidepressant use. Over a 3-year period, the highest risk for incident depression was again observed for those with metabolically unhealthy obesity. For women, significant differences were observed for all three phenotypes in comparison with the metabolically healthy non-obese reference group; in men, differences were significant only between metabolically unhealthy obesity and non-obesity groups (34). However, despite considerable differences in study designs and criteria used for identifying obesity phenotypes and depression, our reported patterns in the obesity phenotype-depression associations are somewhat consistent.

Bidirectional relationships between obesity and depression (5) might be explained, at least in part, by shared genetic, lifestyle and environmental factors and, in the case of metabolically unhealthy individuals, mediated by activated systemic immune-inflammatory and oxidative stress pathways. Consequent dysregulation of these pathways through, for example, lifestyle or medication approaches, may contribute to the onset and progression of depression via hyper-activation of brain inflammatory responses (35). This has been conceptualized as the parallel processes of neuroprogression and somatoprogression of mental health and medical disorders, respectively (36). An extension to our study has the potential to identify behavioral modifications and new treatments for depression that target metabolic disorders. For example, aspects of diet quality such as pro-inflammatory diets could impact cardiometabolic disease (37), body composition (38, 39) and depression (40). Further, new treatments such as insulin sensitizers that affect mitochondrial pathways could disrupt progression to metabolic syndrome, inflammation, oxidative stress (41) and depression, given shared pathways. While our research supports this notion, Mendelian Randomization studies might be needed to tease out the directionality of such relationships (42, 43).

In most studies investigating the obesity-depression relationships, obesity has been defined as high body mass index, which indicates excessive weight-for-height but does not distinguish between different body compositions (44, 45). This may be more problematic in men than women where excess weight might be more attributable to muscular body builds; also, differences become more pronounced at older ages where contributions from fat become higher and muscle lower for a given body mass index compared to younger ages (45). As skeletal muscle is a metabolically active tissue, involuntary loss of muscle mass during aging or periods of immobilization compromises immune system activity and impairs metabolism, and these changes can contribute to the observed relationships between sarcopenia and poor metabolic health. A key strength of our study was assessment by gold-standard techniques used for classifying obesity phenotypes beyond body mass index. The use of DXA allowed us to distinguish fat, lean and bone mass, which is important given some evidence for an association between low muscle mass and depression (46, 47). In sarcopenic obesity, the dual adverse effects of excessive fat and low muscle mass could be overlooked if relying on body mass index as an indicator of unhealthy body composition (13, 48). Evidence of sexual dimorphism in the obesity phenotype-depression relationship (33, 34) might reflect misclassification of obesity phenotyping in men based on body mass index. Our study included women only, and results may not be pertinent to men; comparable research involving men is currently in progress.

Evidence for a relationship between sarcopenic obesity and depression is mixed, and the absence of consensus for defining sarcopenia and sarcopenic obesity is likely contributing to the heterogeneous results in the literature. A recent systematic review reported limited evidence for sarcopenic obesity as a predictor of depressive symptoms; among seven studies that met inclusion criteria, two identified sarcopenic obesity as a predictor of depressive symptoms (49). While a sarcopenic obesity-depression relationship was more likely when measures of muscle strength rather than muscle mass alone were considered when identifying sarcopenia, there were also methodological inconsistencies in how obesity and depression were identified. In one of the included studies, a cross-sectional study of older men and women from Japan, body composition was measured by bioelectric impedance analysis, sarcopenia was defined by low muscle mass and poor physical function, and obesity by high body fat percentage and body mass index. The authors reported that the group with sarcopenic obesity were more likely to have depressive symptoms than those with non-sarcopenia/non-obesity; however, no associations with depressive symptoms were observed for sarcopenia or obesity alone, nor when obesity was defined by high body mass index (50). Further research that considers muscle quality in sarcopenic obesity is needed to account for body compositional changes that occur during aging, particularly sedentary aging, which have the potential to influence the relationship between sarcopenic obesity and depression.

Strengths of our study included the use of the structured clinical interview for the diagnosis of MDD and age of onset, and the use DXA-derived objective measures of body composition for identifying obesity and sarcopenic obesity. However, we identified obesity phenotypes at baseline only and acknowledge that we have not accounted for changes during follow-up that might misclassify individuals as having metabolically healthy obesity, which is recognized as a transient state (12). Nonetheless, obesity tends to be a stable phenotype and is highly resistant to intervention. While our data point to MDD rates over several years in relation to a current obesity phenotype, which is a strength of the study, we acknowledge possible under-estimation of the relationship between obese + highCRP and MDD. While elevated hsCRP values were those in the upper tertile of the distribution in our study population (>2.99 mg/L), we note that this threshold corresponds to that referred to by Chae et al. (>3 mg/L) in a different population (51). We also acknowledge the limitation posed by the use of a single measure of hsCRP to indicate low-grade inflammation, and that this inflammatory biomarker may not embody complex underlying physiological links between immunometabolic dysregulation and depression (31). As the sample comprised women, most of whom were white, the findings may not be generalizable to men or other ethnicities. Last, it was outside the scope of this analysis to explore drivers of inflammation, depression or obesity that might contribute to these results.

## Conclusion

Our study shows that women with metabolically unhealthy obesity characterized by systemic inflammation were at greatest risk for MDD over a 16-year period. These observations suggest that targeting the pro-inflammatory state of obesity, at least among women, might help to reduce the incidence of MDD. Clinicians could be alerted to psychiatric implications of metabolically unhealthy obesity in tandem with systemic inflammation, as appropriate interventions may yield benefits in improving both physical and mental health.

## Data availability statement

The raw data supporting the conclusions of this article will be made available upon reasonable request.

## Ethics statement

This study involving humans was approved by the Human Research Ethics Committee at Barwon Health. The study was conducted in accordance with the local legislation and institutional requirements. The participants provided their written informed consent to participate in this study.

## Author contributions

JP and MB contributed to study conception and design of this study. JP analyzed the data and drafted this manuscript. JP, MB, BP, MK, and LW interpreted the data. All authors contributed to the article and approved the final version.
